# Encephalo-Arterio-Synangiosis with Cranioplasty after Treatment of Acute Subdural Hematoma Associated with Subcortical Hemorrhage Due to Unilateral Moyamoya Disease

**DOI:** 10.1155/2023/1787738

**Published:** 2023-01-17

**Authors:** Naoki Kato, Shota Kakizaki, Yusuke Hirokawa, Shotaro Michishita, Takuya Ishii, Tohru Terao, Yuichi Murayama

**Affiliations:** ^1^Department of Neurosurgery, Atsugi City Hospital, 1-16-36, Mizuhiki, Atsugi, Kanagawa 243-8588, Japan; ^2^Department of Neurosurgery, The Jikei University School of Medicine Tokyo, 3-25-8, Nishi-Shinbashi, Minato-ku, Tokyo 105-8461, Japan; ^3^Department of Neurosurgery, The Jikei University Daisan Hospital, 4-11-1, Izumi-honcho, Komae City, Tokyo 201-8601, Japan

## Abstract

Moyamoya disease is often diagnosed after intracranial hemorrhage in adult patients. Here, we report a case of unilateral moyamoya disease treated with indirect revascularization combined with cranioplasty after treatment for acute subdural hematoma and subcortical hemorrhage. A middle-aged woman with disturbed consciousness was transferred to our hospital. Computed tomography (CT) revealed an acute subdural hematoma with left temporoparietal subcortical hemorrhage. Three-dimensional CT angiography indicated a scarcely enhanced left middle cerebral artery (MCA) that was suspected to be delayed or nonfilling due to increased intracranial pressure. Subsequently, hematoma evacuation and external decompression were performed. Postoperative digital subtraction angiography (DSA) revealed stenosis of the left MCA and moyamoya vessels, indicating unilateral moyamoya disease. Forty-five days after the initial procedure, we performed encephalo-arterio-synangiosis (EAS) using the superficial temporal artery simultaneously with cranioplasty for the skull defect. The modified Rankin Scale score of the patient one year after discharge was 1, and the repeat DSA showed good patency of the EAS. Revascularization using EAS in the second step can be an option for revascularization for hemorrhagic moyamoya disease if the patient required cranioplasty for postoperative skull defect after decompressive craniotomy.

## 1. Introduction

Moyamoya disease is a known cause of intracerebral hemorrhage in adult patients, and acute subdural hematoma associated with moyamoya disease sometimes requires decompressive craniotomy, as reported in previous studies [1, 2]. Recently, surgical revascularization has been established as an effective treatment for moyamoya disease in order to reduce recurrent strokes [1, 3]. We report a case of unilateral moyamoya disease with acute subdural hematoma, in which simultaneous encephalo-arterio-synangiosis (EAS) and cranioplasty were successfully performed in the subacute phase.

## 2. Case Presentation

**Figure 1:**
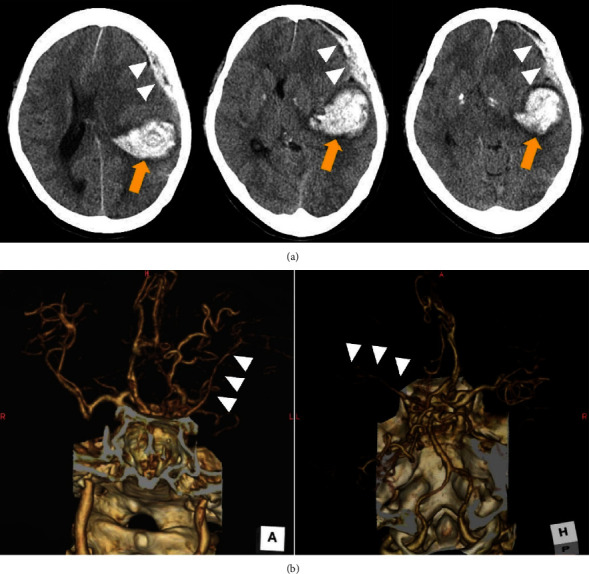
Preoperative radiological examinations. (a) Computed tomography (CT) showed subcortical temporoparietal hemorrhage (arrow) and subdural hematoma (arrowheads) with midline shift of the cerebrum as well as uncal herniation. (b) Three-dimensional CT angiography indicated scarcely enhanced the left middle cerebral artery (arrow heads): (left) anterior-posterior and (right) posterior-anterior view.

**Figure 2:**
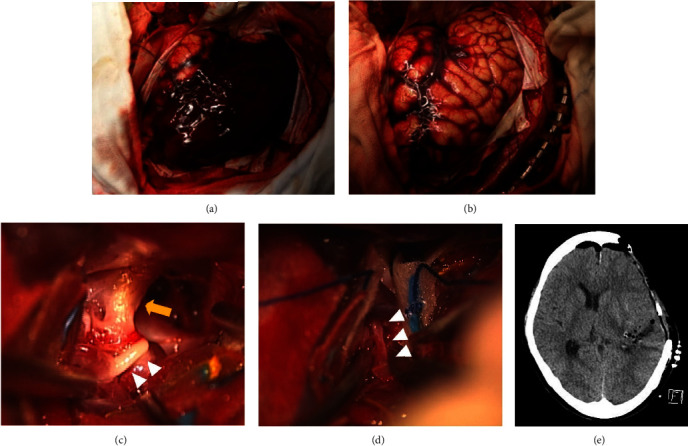
Images of the initial procedure. (a) Photograph showing a huge subdural hematoma after opening the dura mater. (b) Photograph after evacuation of the hematoma showing a swollen brain surface. (c) Intraoperative microscopic photograph showing the left internal carotid artery (arrow) and M1 segment of the hypoplastic left middle cerebral artery (MCA) (arrowheads). (d) Intraoperative microscopic showing a narrow M2 segment of the hypoplastic left MCA (arrowheads). (e) Computed tomography immediately after surgery, confirming adequate decompression and removal of the hematoma.

**Figure 3:**
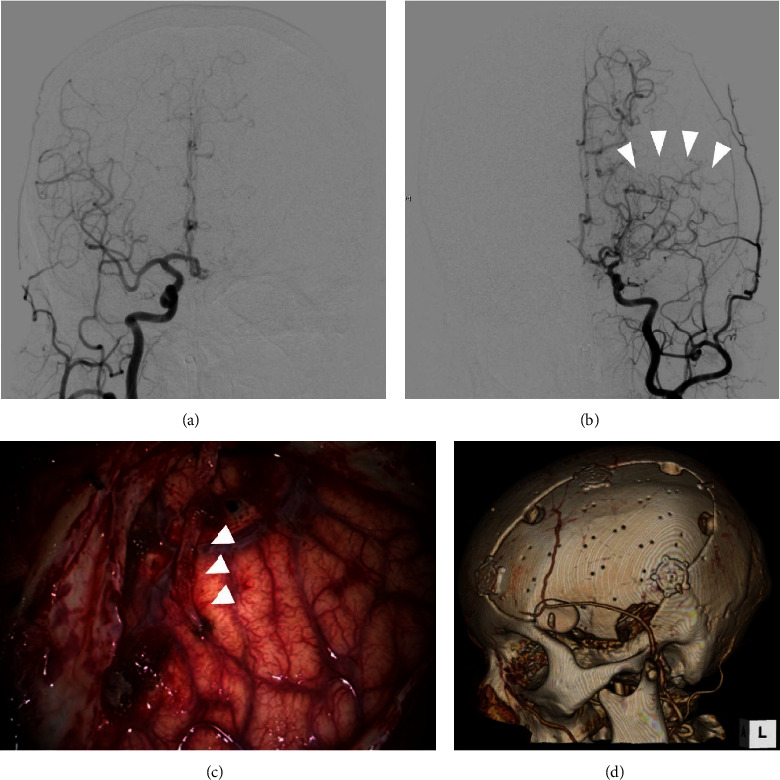
Images after initial procedure. (a) Digital subtraction angiography (DSA) of the right common carotid artery (CCA) after the initial surgery showing no remarkable abnormality. (b) DSA of the left CCA showed stenosis of the middle cerebral artery and moyamoya vessels (arrowheads). (c) Intraoperative photograph demonstrating encephalo-arterio-synangiosis between the brain surface and the parietal branch of the superficial temporal artery (STA) (arrowheads). (d) Three-dimensional computed tomography after the second surgery showing the structure of the implanted bone flap and STA.

A middle-aged woman with sudden consciousness disturbance was transferred to our institute. Initially, her Glasgow Coma Scale score was 4, and anisocoria and decorticate position of extremities were observed. Computed tomography (CT) demonstrated an acute subdural hematoma associated with left temporoparietal subcortical hemorrhage (Figure 1(a)). Three-dimensional CT angiography (3DCTA) indicated a round shift of the bilateral anterior cerebral artery and a scarcely enhanced left middle cerebral artery (MCA) (Figure 1(b)). We expected that the decreased contrast medium in the left MCA was the result of delayed- or non-filling of the contrast medium due to increased intracranial pressure of the ipsilateral hemisphere. Subsequently, we performed external decompression and hematoma evacuation (Figures 2(a) and 2(b)). The superficial temporal artery (STA) was preserved upon scalp incision. During the procedure, we could not identify any aneurysms or arteriovenous malformations but observed marked stenosis of the MCA vessels, indicating hypoplastic MCA of the ipsilateral side (Figures 2(c) and 2(d)). Adequate evacuation of the hematoma and decompression was confirmed on postoperative CT (Figure 2(e)). Postoperative digital subtraction angiography (DSA) three days after the initial surgery revealed severe stenosis of the MCA and moyamoya vessels on the left side; therefore, we diagnosed the patient with unilateral moyamoya disease (Figures 3(a) and 3(b)). Approval of data use regarding the treatment, images for research, and academic presentation was obtained from the patient.

### 2.1. Treatment

As a treatment for unilateral moyamoya disease to reduce the stroke recurrence, we planned to conduct surgical revascularization at the same step as cranioplasty for the postoperative skull defect. Forty-five days after the initial surgery, we performed a combined procedure. In the first step, we attempted direct revascularization between the parietal branch of the STA and the MCA. However, because of the adhesion of surrounding tissues and the small size of recipient arteries, it was difficult to achieve successful direct anastomosis. We then decided to perform indirect revascularization. We simply placed the end of the STA onto the surface of the M4 segment of the MCA with sutures using 10-0 nylon (Figure 3(c)). After indirect revascularization, cranioplasty using the patient's skull flap was performed (Figure 3(d)).

### 2.2. Outcome and Follow-Up

**Figure 4:**
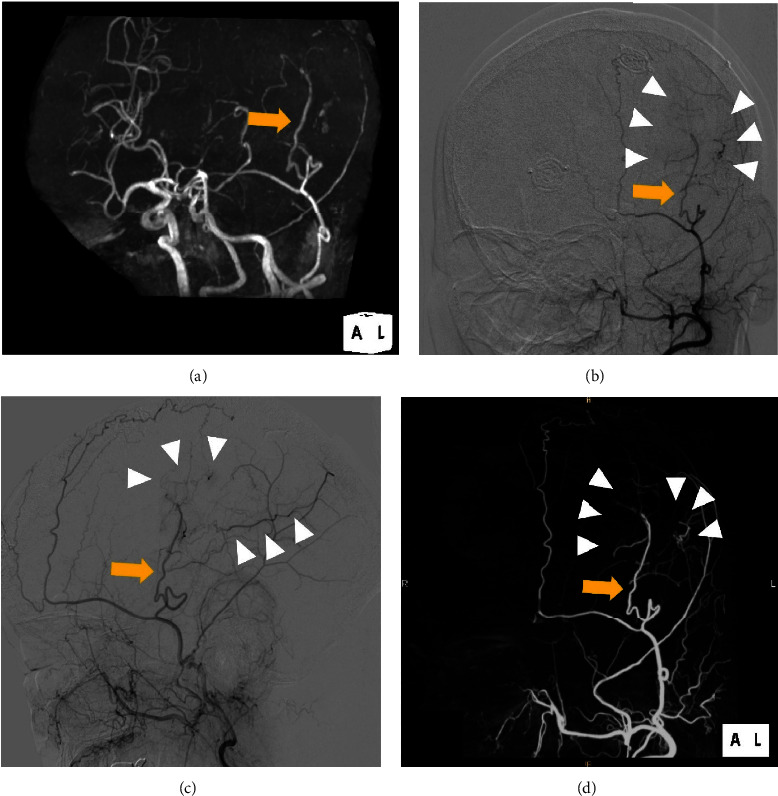
Radiological findings after the second surgery. (a) Repeat magnetic resonance angiography showing the patent vessel of encephalo-arterio-synangiosis (arrow). The patent superficial temporal artery (STA) (arrow) and revascularized M4 segment of the middle cerebral artery (MCA) (arrow heads) were confirmed by digital subtraction angiography (DSA) of the external carotid artery (ECA); (b) left anterior-oblique and (c) lateral view. Three-dimensional DSA of the ECA verified good patency of the parietal branch of the STA (arrow) and the collateralized MCA (arrow heads); (d) left anterior-oblique view.

The postoperative course was uneventful, and her symptoms gradually improved. The modified Rankin Scale score of the patient one year after the treatment was 1, and regular magnetic resonance angiography (MRA) indicated increased blood flow in the revascularized STA (Figure 4(a)); there was no recurrence of stroke. Repeat DSA three years after the treatment showed good patency of the indirectly anastomosed STA and collateral formation, which was evaluated as “B” score of the Matsushima scale; with A being more than 2/3 of the MCA distribution, B in between 2/3 and 1/3 of the MCA distribution, and C being slight or none (Figures 4(b)–4(d)) [4].

## 3. Discussion

In this case report, we describe the case of a patient with unilateral moyamoya disease who presented with an acute subdural hematoma associated with subcortical hemorrhage. According to previous studies, moyamoya disease might cause severe intracranial bleeding leading to acute subdural hematoma [5–8]. Before the initial surgical treatment, we suspected that the invisible ipsilateral MCA on 3DCTA was due to the delayed- or non-filling phenomenon of contrast medium due to severely increased intracranial pressure, which may predict an unfavorable prognosis [9, 10]. However, the postoperative examination revealed unilateral moyamoya disease that mimicked the delayed- or non-filling phenomenon, and the prognosis was relatively favorable. Although 3DCTA is a useful investigation modality in emergent cases for detecting aneurysms or vascular malformations, it should be considered that it may not be able to adequately distinguish moyamoya disease or MCA stenosis from the delayed- or non-filling phenomenon in the acute phase.

Recently, several articles have stated that surgical revascularization has the potential to reduce the risk of recurrent stokes in patients with moyamoya disease [1, 11]. Specifically, the Japan Adult Moyamoya Disease Trial presented that STA-MCA bypass combined with indirect revascularization may prevent secondary hemorrhage in patients suffering from hemorrhagic moyamoya disease [1]. Indirect revascularization with or without STA-MCA bypass, such as encephalo-duro-arterio-synangiosis (EDAS), encephalo-duro-myo-arterio-synangiosis (EDMAS), or encephalo-duro-myo-arterio-pericranial synangiosis (EDMAPS), is used in children or young patients with moyamoya disease [12–14]. In addition, indirect revascularization may be effective in adult patients with moyamoya disease as well [15]. Hee Han et al. reported that indirect revascularization, i.e., EDAS, can improve the clinical outcomes of patients with moyamoya disease if the onset was due to bleeding [15]. Another large case series reported that anterior hemorrhage was associated with better postoperative collateral formation in moyamoya disease [16]. In the present case, we could not achieve direct anastomosis and carried out indirect bypass, i.e., EAS, during the procedure for cranioplasty. At the first treatment, we could not anticipate the cause of the hemorrhage to be unilateral moyamoya disease or the necessity for subsequent revascularization since we considered the findings of the 3DCTA to be the delayed or nonfilling phenomenon due to the large hematoma on the ipsilateral side. However, careful preservation of the STA during the decompressive craniotomy was unexpectedly helpful for the EAS at the second step. Some articles have reported that indirect anastomosis between the STA and the cerebral surface through a small craniotomy can lead to marked revascularization after surgery [13]. Even if direct anastomosis could not be achieved after the surgery, the anastomosed graft has the potential to form a patent anastomosis [17]. In the present case, direct bypass was relatively difficult due to repeated craniotomy, and we decided to perform indirect bypass using the STA. At this time, we did not use the dura mater or temporal muscle for the synangiosis because the adhesion under the dura mater was severe, and we prioritized a certain placement of the patient's skull as well as better healing of repeated scalp incision. However, other indirect revascularization techniques, such as EDAS or EDMAS, could be considered in this situation for maximizing the collateral formation. Fortunately, the graft gradually became patent, similar to a directly anastomosed vessel. Although the present case did not undergo postoperative imaging regarding cerebral blood flow, evaluation using single photon emission CT or perfusion CT after the EAS should have been taken into account. It may be able to assess the positive effect of the synangiosis. We believe that simultaneous indirect bypass and cranioplasty can be a viable treatment option for patients with moyamoya disease after severe intracranial bleeding.

This case of hemorrhagic moyamoya disease mimicked the delayed or nonfilling phenomenon and was successfully treated with the step-by-step surgical treatment including indirect revascularization. We found that EAS combined with cranioplasty may be a viable option for the second procedure.

## Data Availability

No data were used to support the findings of this study.
